# Comparison between blue-on-yellow and white-on-white perimetry in patients with branch retinal vein occlusion

**DOI:** 10.1038/s41598-020-77025-x

**Published:** 2020-11-17

**Authors:** Kunihiro Azuma, Tatsuya Inoue, Ryosuke Fujino, Nozomi Igarashi, Shotaro Asano, Yoko Nomura, Yohei Hashimoto, Keiko Azuma, Ryo Asaoka, Kazuaki Kadonosono, Ryo Obata

**Affiliations:** 1 Department of Ophthalmology, The University of Tokyo, Tokyo, Japan; 2 Department of Ophthalmology and Micro-Technology, Yokohama City University, 4-57 Urafune, Minami-ku, Yokohama, 232-0024 Japan; 3 Department of Ophthalmology, Seirei Hamamatsu General Hospital, Shizuoka, Hamamatsu Japan; 4 Seirei Christopher University, Shizuoka, Hamamatsu Japan

**Keywords:** Medical research, Optics and photonics, Eye diseases, Retinal diseases

## Abstract

This study aimed to compare blue-on-yellow (B/Y) perimetry with white-on-white (W/W) perimetry in eyes with branch retinal vein occlusion (BRVO). The following measurements were performed in 29 eyes of 29 patients with resolved BRVO: W/W and B/Y perimetries using 10-2 test grid, retinal volume (RV) using optical coherence tomography (OCT), and vessel densities (VD) of the superficial capillary layer (VDs) and deep capillary layer (VDd) using OCT angiography (OCTA). First, the difference in the retinal sensitivity (RS) between BRVO-affected and unaffected areas was compared between RS_B/Y and RS_W/W in the parafoveal and extrafoveal areas. Moreover, the structure–function relationship between vessel density and RS was compared between B/Y and W/W perimetries (RS_B/Y and RS_W/W, respectively). The difference in RS between BRVO-affected and unaffected areas was significantly larger with RS_B/Y than with RS_W/W in both the parafoveal and extrafoveal areas. In the parafoveal area, VDs, VDd, and RV were significantly correlated with both RS_W/W and RS_B/Y. In contrast, in the extrafoveal area, only VDd was included in the optimal models. Our findings suggest that RS_B/Y more strongly reflects the anatomical structure and BRVO-affected area.

## Introduction

Branch retinal vein occlusion (BRVO) is one of the common retinal vascular diseases in the clinical setting. Eyes affected with BRVO often develop a retinal capillary nonperfused area (NPA) that may lead to neovascularization and vitreous hemorrhage^1^. Fluorescein angiography has been the gold standard to detect NPA; however, more detailed evaluation of the retinal vessels is now possible using the recently developed method of optical coherence tomography angiography (OCTA)^2^. The superficial and deep retinal vasculatures can be observed separately using the *en face* visualization technique^3^. Recent studies have revealed that the retinal vessels are associated with visual function in eyes with BRVO. In a study by Wakabayashi et al., the microvasculature of the vascular perfusion area in the deep capillary plexus within a 3 × 3-mm macular area was found to be strongly correlated with visual acuity in eyes with resolved BRVO^4^. Another study suggested that there is a significant reduction in retinal sensitivity (RS) in NPA measured using OCTA in eyes with BRVO^5^.

Blue-on-yellow (B/Y) perimetry uses a short-wavelength blue stimulus on a high-luminance yellow background that can be used to evaluate the function of blue cones by isolating them from the other cones. Early detection of glaucomatous visual field (VF) damage is one of the most representative merits of B/Y perimetry compared with the conventional white-on-white (W/W) perimetry^6–8^. Furthermore, B/Y perimetry has reportedly been advantageous in detecting early abnormalities in many fundus diseases, such as diabetic retinopathy, optic nerve disorders, and central serous chorioretinopathy^9–13^. However, to the best of our knowledge, there have been few studies on BRVO using B/Y perimetry.

Therefore, this study aimed to compare B/Y perimetry and W/W perimetry in eyes with BRVO and perform a detailed analysis of the structure–function relationship between vessel density (VD) and RS using both perimetries.

## Results

A total of 29 eyes of 29 patients with resolved BRVO were enrolled in this study. Baseline characteristics of the subjects are presented in Table 1. All the patients had hypertension but not hyperlipidemia. There were 17 eyes with major BRVO and 12 with macular BRVO.

**Table 1:** Baseline characteristics of the patients.

Variable	Mean ± SD	Range
Eyes (phakia/pseudophakia)	29 (23/6)	
Age (years)	68.3 ± 9.53	52–85
Gender (male to female)	11:18	
logMAR VA	0.057 ± 0.18	− 0.079 to 0.82
Mean retinal sensitivity in W/W perimetry (dB)	29.58 ± 2.1	25.1–32.92
Mean retinal sensitivity in B/Y perimetry (dB)	23.11 ± 3.77	17.15–29.27
CRT (μm)	268.17 ± 33.57	197–364
CCT (μm)	171.55 ± 34.70	75–238

*logMAR VA* logarithm of the minimum angle resolution visual acuity, *CRT* central retinal thickness, *CCT* central choroidal thickness.

The values of the retinal sensitivity measured using W/W and B/Y perimetries (RS_W/W and RS_B/Y, respectively), VD in the superficial capillary plexus (SCP) and deep capillary plexus (DCP) measured using OCTA (VDs and VDd, respectively), and retinal volume (RV) are presented in Table 2. The correlation between functional measurement and OCT parameters was analyzed using 6-mm Early Treatment of Diabetic Retinopathy Study (ETDRS) grid. Three eyes had central macular nonperfusion, and the other 26 eyes had central macular perfusion. The representative image of an eye with central macular nonperfusion is shown in Supplementary Figure S1.

**Table 2:** Mean value of each measurement.

	RS_W/W (dB)	RS_B/Y (dB)	VDs (%)	VDd (%)	RV (mm^3^)
Parafovea	30.15 ± 3.19 (11.5–34.25)	23.54 ± 5.25 (2.17–32)	81.41 ± 6.79 (61.40–95.71)	86.68 ± 8.25 (60.88–98.92)	0.5 ± 0.05 (0.31–0.63)
Extrafovea	28.82 ± 3.48 (14.50–33.10)	22.85 ± 5.17 (1.33–30.90)	77.47 ± 6.56 (59.70–92.88)	81.75 ± 8.78 (50.00–98.14)	1.53 ± 0.12 (1.23–1.88)
Center	30.37 ± 2.87 (19.75–33.75)	22.37 ± 4.99 (3.75–28.50)	67.68 ± 6.12 (54.49–78.59)	63.86 ± 7.19 (46.42–74.67)	0.21 ± 0.03 (0.15–0.29)
Total	29.58 ± 2.1 (25.09–32.92)	23.11 ± 3.77 (13.19–29.59)	78.13 ± 4.04 (69.59–84.25)	81.93 ± 5.42 (69.46–90.27)	0.93 ± 0.05 (0.82–1.02)

*RS_W/W* retinal sensitivity in white-on-white perimetry, *RS_B/Y* retinal sensitivity in blue-on-yellow perimetry, *VDs* vessel density in superficial capillary plexus, *VDd* vessel density in deep capillary plexus, *RV* retinal volume.

The results of the univariate analysis between the best-corrected visual acuity (BCVA) and other parameters in the center and parafovea are presented in Supplementary Table S1. No parameters were significantly related to BCVA in the central area. In the parafovea, VDs and RV were significantly correlated to BCVA (linear model, *P* = 0.037 and *P* = 0.011, respectively). Based on the second-order bias-corrected Akaike Information Criterion (AICc) model selection for BCVA, only the parafoveal RV was selected as the explanatory variable (AICc =  − 16.3).

Results of univariate analyses and the result of AICc model selection between RS and other parameters in the central, parafoveal, and extrafoveal areas are presented in Table 3. In the central area, no parameters were significantly related to RS using both perimetries.

**Table 3:** Correlation between retinal sensitivity measured using both perimetries and other parameters.

	Univariate				Multivariate		
	Variables	Estimate	SE	*P* value	Estimate	SE	*P* value
Parafovea RS_W/W	Age	− 0.068	0.036	0.067	N.S	N.S	N.S
	VDs	0.25	0.039	< 0.001	N.S	N.S	N.S
	VDd	0.24	0.029	< 0.001	0.19	0.032	< 0.001
	RV	28.09	4.91	< 0.001	14.18	4.86	0.004
Parafovea RS_B/Y	Age	− 0.11	0.067	0.10	N.S	N.S	N.S
	VDs	0.38719	0.064	< 0.001	N.S	N.S	N.S
	VDd	0.40	0.046	< 0.001	0.33	0.05	< 0.001
	RV	46.15	8.09	< 0.001	23.6	7.71	0.002
Extrafovea RS_W/W	Age	− 0.070	0.046	0.14	N.S	N.S	N.S
	VDs	0.11	0.047	0.022	N.S	N.S	N.S
	VDd	0.12	0.037	0.001	0.12	0.037	0.002
	RV	5.63	2.45	0.024	N.S	N.S	N.S
Extrafovea RS_B/Y	Age	− 0.11	0.073	0.14	N.S	N.S	N.S
	VDs	0.18	0.066	0.007	N.S	N.S	N.S
	VDd	0.22	0.052	< 0.001	0.22	0.052	< 0.001
	RV	8.72	3.42	0.012	N.S	N.S	N.S
Center RS_W/W	Age	− 0.026	0.058	0.66	N.S	N.S	N.S
	VDs	0.060	0.089	0.51	N.S	N.S	N.S
	VDd	0.084	0.075	0.27	N.S	N.S	N.S
	RV	− 5.31	20.08	0.79	N.S	N.S	N.S
Center RS_B/Y	Age	− 0.059	0.10	0.56	N.S	N.S	N.S
	VDs	0.16	0.15	0.30	N.S	N.S	N.S
	VDd	0.042	0.13	0.76	N.S	N.S	N.S
	RV	27.50	34.54	0.43	N.S	N.S	N.S

*SE* standard error, *RS_W/W* retinal sensitivity in white-on-white perimetry, *RS_B/Y* retinal sensitivity in blue-on-yellow perimetry, *VDs* vessel density in superficial capillary plexus, *Dd* vessel density in deep capillary plexus, *RV* retinal volume, *N.S*. not selected (linear mixed model, model selection using the second-order bias-corrected Akaike Information Criterion index).

In the parafoveal area, VDs, VDd, and RV were significantly correlated with RS_W/W and RS_B/Y (both *P* < 0.001, linear mixed model). In the extrafoveal area, VDs, VDd, and RV were also correlated with RS_W/W and RS_B/Y (linear mixed model*, P* = 0.02, *P* = 0.001, and *P* = 0.02 with W/W perimetry; *P* = 0.007, *P* < 0.001, and *P* = 0.01 with B/Y perimetry, respectively). AICc model selection was conducted for RS_W/W and RS_B/Y. The optimal model for RS_W/W and RS_B/Y in the parafoveal area was as follows: RS_W/W = 6.52 + 0.18 (± 0.03) × VDd + 15.45 (± 4.63) × RV (AICc = 539.9) and RS_B/Y =  − 17.03 + 0.32 (± 0.05) × VDd + 25.42 (± 7.12) × RV (AICc = 635.4) (Table 3). Conversely, in the extrafoveal area, only VDd was included in the optimal models:

$$\begin{gathered} {\rm{RS}}\_{\rm{W}}/{\rm{W }} = { 18}.{19 } + 0.13( \pm 0.0{39}) \times {\rm{ VDd }}\left( {{\rm{AICc }} = {602.3}} \right) \hfill \\ {\rm{RS}}\_{\rm{B}}/{\rm{Y }} = { 1}.{94 } + 0.{26 }( \pm 0.0{54}) \times {\rm{ VDd }}\left( {{\rm{AICc }} = {675}.{4}} \right) \hfill \\ \end{gathered}$$

**Figure 1:**
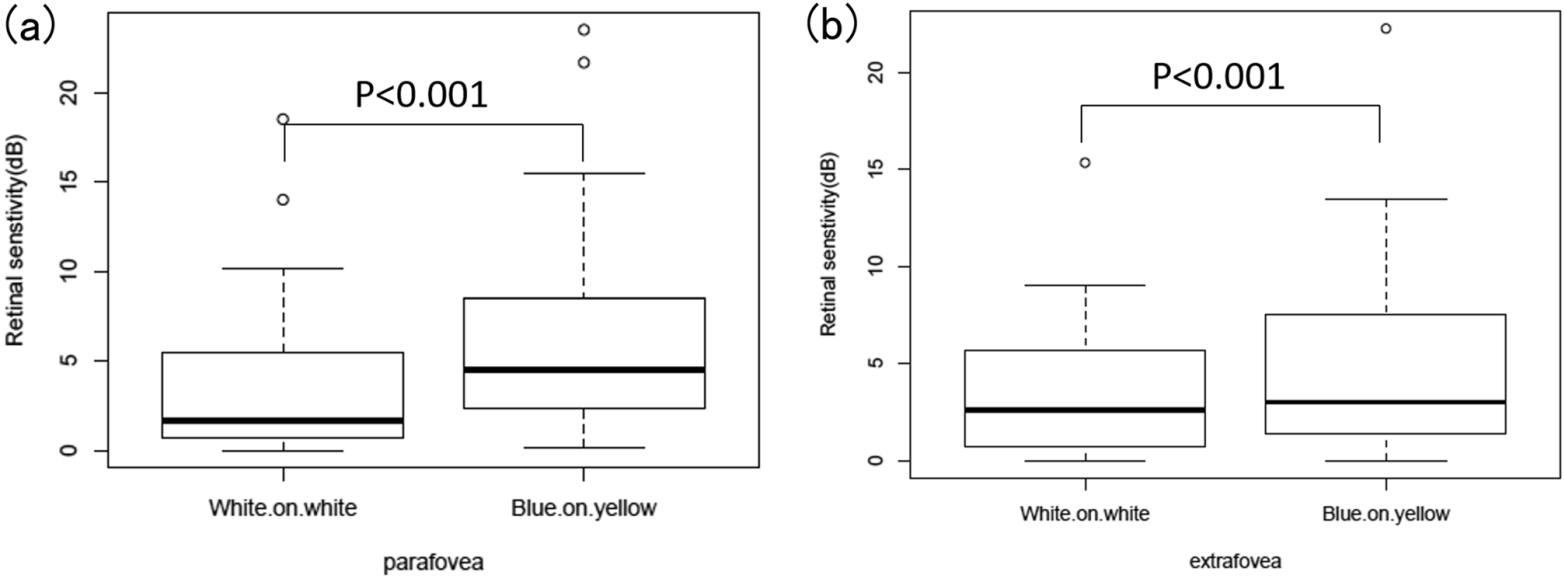
Comparison of the difference in RS between the BRVO-affected and unaffected areas using both perimetries. (**a**) Comparison in the parafovea, (**b**) comparison in the extrafovea. The difference with B/Y perimetry was significantly larger than that with W/W perimetry in both regions (the paired Student’s *t* test). The images were generated using R software version 3.5.2, following the guideline (https://cran.r-project.org/doc/FAQ/R-FAQ.html#Citing-R). *RS* retinal sensitivity, *BRVO* branch retinal vein occlusion, *B/Y* blue on yellow, *W/W* white on white.

The difference in RS between the BRVO-affected and unaffected areas using both perimetries is presented in Fig. 1. The difference in RS using B/Y perimetry was significantly larger than that using W/W perimetry in both the parafoveal and extrafoveal areas (paired *t*-test, *P* < 0.001 and *P* = 0.008, respectively). The result of AICc model selection for structural parameters is presented in Table 4. In both the parafovea and extrafovea, only RS_B/Y was included in the optimal model for VDd, with RS_W/W not being included (AICc = 758.1 and AICc = 798.9, respectively); inclusion of RS_W/W resulted in an increase in AICc values. In the parafovea as well, only RS_B/Y was included in the optimal model for RV (AICc =  − 384.9) and RS_W/W was not.

**Table 4:** AICc model selection evaluating parameters that are important for VDd and RV.

	Variables	Estimate	Standard error	*P* value
Parafovea VDd	RS_W/W	N.S	N.S	N.S
	RS_B/Y	0.99	0.11	< 0.001
Parafovea RV	RS_W/W	N.S	N.S	N.S
	RS_B/Y	0.0045	0.00084	< 0.001
Extrafovea VDd	RS_W/W	N.S	N.S	N.S
	RS_B/Y	0.58	0.15	< 0.001

*AICc* second-order bias-corrected Akaike Information Criterion, *RS_W/W* retinal sensitivity in white-on-white perimetry, *RS_B/Y* retinal sensitivity in blue-on-yellow perimetry, *VDd* vessel density in deep capillary plexus, *RV* retinal volume (linear mixed model, model selection using AICc).

## Discussion

In this study, RS was measured using the B/Y and W/W perimetries in eyes with resolved BRVO, along with measurements obtained using OCTA. Our results suggested that RS measured using B/Y perimetry had a tighter correlation with VDd and RV than RS measured using W/W perimetry. In addition, the results suggested that the difference in RS between the affected and unaffected areas in eyes with BRVO was larger with B/Y perimetry than with W/W perimetry. Taken together, our results suggest that retinal function in eyes with BRVO is better reflected with B/Y perimetry than with W/W perimetry.

This study showed that RV in the parafovea was the most important variable for BCVA, whereas VDd and VDs in the parafoveal area were not. Previous studies have found contradicting results on the association between BCVA and OCT parameters. A recent study has suggested that foveal avascular zone (FAZ) is correlated to VA in both eyes of patients with diabetic retinopathy and in eyes with BRVO^14^. In contrast, Kadomoto et al.^15^ have reported that FAZ is not correlated with VA, but parafoveal NPA in the DCP is strongly associated with VA and macular sensitivity. Wakabayashi et al.^4^ have reported that FAZ, microvascular abnormalities, and VD are related to visual function and that VD of DCP significantly correlates with BCVA measured using OCTA.

The reason behind this discrepancy is unknown, but it is attributable to the difference in data collection. Kadomoto et al. measured parafoveal NPA within 3 × 3 mm in each eye; Wakabayashi et al. measured retinal perfusion area within 3 × 3 mm, whereas we measured VD by dividing the parafoveal region into four parts.

However, when we calculated VD and RS within the total 3 × 3-mm area, only RV was negatively correlated to the logarithm of the minimum angle resolution (logMAR) VA (linear regression, *P* = 0.015) and VDd was not. Conversely, among RV, VDd, and VDs, only VDd was selected as an explanatory variable of RS_W/W and RS_B/Y (linear regression,* P* < 0.0001, respectively).

The present study demonstrated that VDd is an explanatory variable of RS in both perimetries but VDs is not. Previous studies have reported that microvascular abnormalities, such as capillary telangiectasia, microaneurysm, collateral vessels, and FAZ enlargement, are more frequently observed in DCP than in SCP^16–21^. Moreover, Birol et al. have reported that capillary deep layer provides 10–15% of oxygen supply to the photoreceptor cells^22^; hence, ischemia in DCP may gradually influence photoreceptor integrity^23,24^, which is significantly associated with visual outcomes of BRVO^25,26^. Based on the results of this study and previous studies, it is suggested that the perfusion status of DCP compared with that of SCP is more important for RS.

In this study, RS measured with B/Y perimetry compared with that measured with W/W perimetry was more closely associated with VDd and RV. In addition, the difference in RS between the BRVO-affected and unaffected areas was significantly larger with B/Y perimetry than with W/W perimetry, which may be because compared with the long- and medium-wavelength-sensitive cones, the short-wavelength-sensitive cones and their neural connections are more vulnerable to damage caused by lack of oxygen^27–29^. Indeed, previous clinical studies have reported that abnormality with B/Y perimetry occurs at the early stage of diabetic retinopathy than that with W/W perimetry, which is considered as subclinical retinal hypoxia secondary to hyperglycemia^12,30–33^. Smith et al. also reported that experimental hypoxia preferentially led to blue–yellow abnormality in a psychophysiological test using the FM 100-hue test^34^. Our result is attributable to the fact that the number of short-wavelength-sensitive cones is much smaller than that of long- and medium-wavelength-sensitive cones in the macular region.

In the real-world setting, functional assessment of BRVO is usually performed on the basis of BCVA, with perimetric examinations being only rarely conducted. In contrast, several previous studies have suggested the usefulness of measuring RS using W/W perimetry in eyes with BRVO. For instance, some studies have suggested the usefulness of this approach in detecting early and subtle functional damage in eyes with BRVO^5,35–37^. In addition, clinically, it is often observed that patients with resolved BRVO describe visual symptoms such as decreased RS, metamorphopsia, and central scotoma that cannot be fully explained solely on the basis of BCVA. The present study suggests a further greater merit on the use of B/Y perimetry in measuring such pathologies in eyes with BRVO.

This study has several limitations. First, the study has a retrospective nature, and the number of examined eyes was relatively small. Second, to compare 6-mm ETDRS grid, we measured VF only within 10°, and VF did not cover the whole BRVO-affected area. Evaluation of RS in a wider area may be further advantageous to assess the structure–function relationships in BRVO.

In conclusion, we found that VDd and RV are more strongly correlated to RS_B/Y than to RS_W/W. Moreover, the difference in RS between BRVO-affected and unaffected areas was significantly larger with B/Y perimetry than with W/W perimetry. These findings indicate that it is useful to assess RS using B/Y perimetry in eyes with BRVO.

## Methods

This study was approved by the Research Ethics Committee of the Graduate School of Medicine and Faculty of Medicine at The University of Tokyo. Written informed consent was obtained from the patients for their information to be stored in the hospital database and used for research. The study protocol was in accordance with the Declaration of Helsinki.

### Subjects

The study included 29 eyes (15 right and 14 left eyes) of 29 patients with resolved BRVO, who were examined at the Department of Ophthalmology of the University of Tokyo Hospital. All eyes had received antivascular endothelial growth factor therapies. Macular edema and serous retinal detachment were completely resolved as shown by careful examination using OCT macular scans. Eyes with other ophthalmic diseases, such as epiretinal membrane, glaucoma, and diabetic retinopathy, and eyes that had undergone photocoagulation therapy were excluded. Eyes with cataract, which could hinder VF measurement, were excluded.

All patients had undergone comprehensive ophthalmologic examinations such as BCVA, slit-lamp biomicroscopy, intraocular pressure, and indirect fundus ophthalmoscopy. In addition, measurements using W/W perimetry, B/Y perimetry, OCT, and OCTA were performed the same day, as detailed below.

### Visual field test

Each patient underwent W/W and B/Y VF testing using AP-7000 (Kowa, Tokyo, Japan). Both VF test measurements were performed applying the 10-2 test grid using the Goldmann size III target. W/W and B/Y perimetry measurements were performed in a random order. Both VF tests were performed using the Quick 1 algorithm, which reportedly has outcomes similar to those obtained using the Swedish interactive threshold algorithm standard test^38^. B/Y perimetry was performed under a 450-nm narrow band target on a bright broad band 100 cd/m^2^ against a yellow background (600 nm). We confirmed that all of the VF measurements satisfied the reliability criteria, defined as a fixation loss rate of < 20%, false-positive rate of < 15%, and false-negative rate of < 33%.

### OCT measurement

All the patients underwent macular topographic mapping under raster scan protocol using Spectral Domain OCT (SPECTRALIS OCT, Heidelberg Engineering, Heidelberg, Germany). The raster scan was performed using 25 B-scans (768 A-scans per B-scan) of a 30° × 20° area. Analysis of RV with a 6-mm ETDRS grid was automatically performed using SPECTRALIS OCT built-in software.

### OCTA measurement and vessel density

#### OCTA was performed using Avanti RTVue XR (Optovue, Fremont, CA)

**Figure 2:**
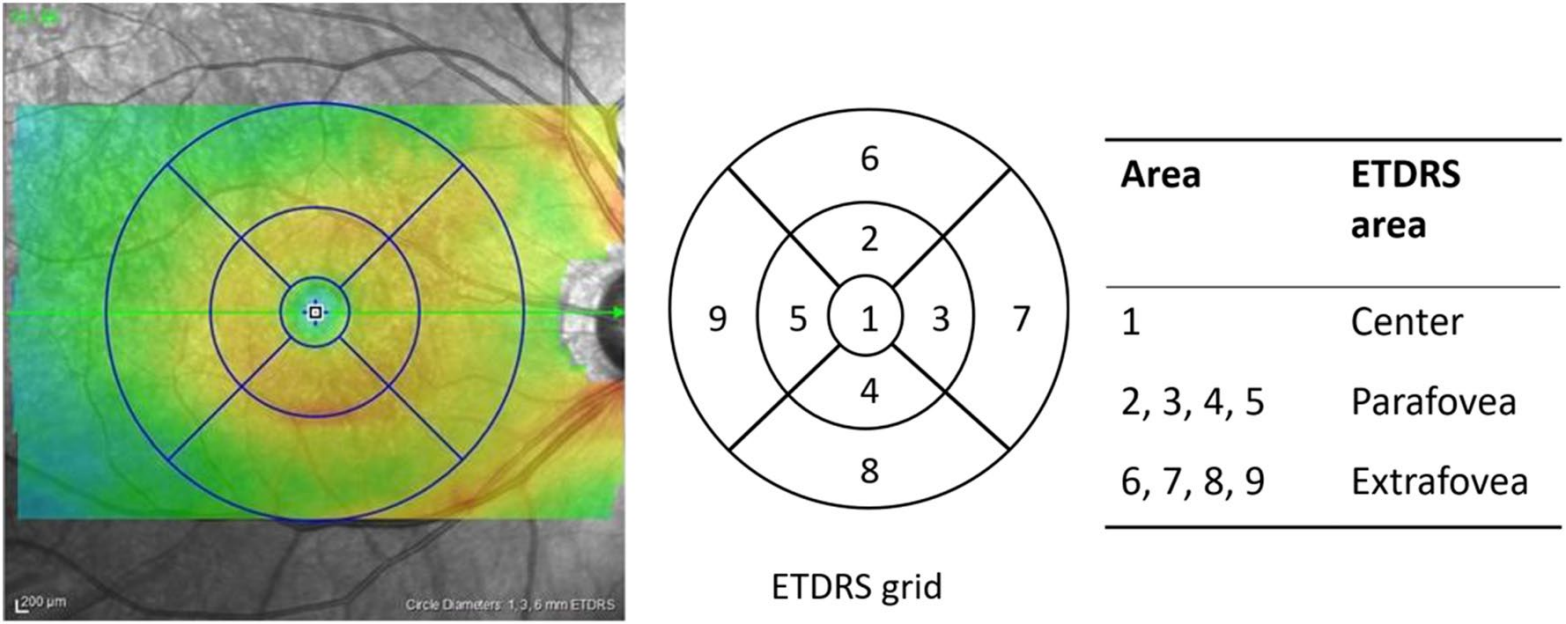
Sectorization of the macula. The built-in OCT software automatically analyzed the mean retinal thickness and volume in each of the nine macular regions in a 6-mm-diameter circle that was centered on the fovea, as defined in ETDRS. The diameter of the central circle was 1 mm, that of the inner ring was 3 mm, and that of the outer ring was 6 mm. The center circle was defined as the center, the inner ring as the parafovea, and the outer ring as the extrafovea. *VF* visual field, *ETDRS* Early Treatment of Diabetic Retinopathy Study.

The scanning area was captured in 6 × 6-mm sections centered on the fovea. To obtain images of SCP, the SCP slab was taken from the internal limiting membrane (offset, 3 μm) to the inner plexiform layer (offset, 15 μm). To obtain images of DCP, the DCP slab was taken from the inner plexiform layer (offset, 15 μm) to the outer plexiform layer (offset, 70 μm). To evaluate the capillary perfusion status of SCP and DCP, binarized *en face* images were prepared using ImageJ software (ImageJ, V.2.0.0-rc-69/1.52i, NIH, Bethesda, Maryland, USA). A modified Niblack’s method was used to perform binarization of each *en face* image, similar to our previous study^39^. Briefly, the image was converted to 8 bits and adjusted using the Niblack auto local threshold. Then, we set the regions of interest that were each of the nine macular regions in a 6-mm-diameter circle that was centered on the fovea, as defined in ETDRS (Fig. 2). We defined the center circle of 1-mm diameter on the fovea as the center, a 3-mm-diameter concentric circle as the parafovea, and a 6-mm-diameter concentric circle as the extrafovea. The mean gray value was calculated for each of the nine macular regions and was defined as VD.

### Mapping VF to OCT/OCTA images

**Figure 3:**
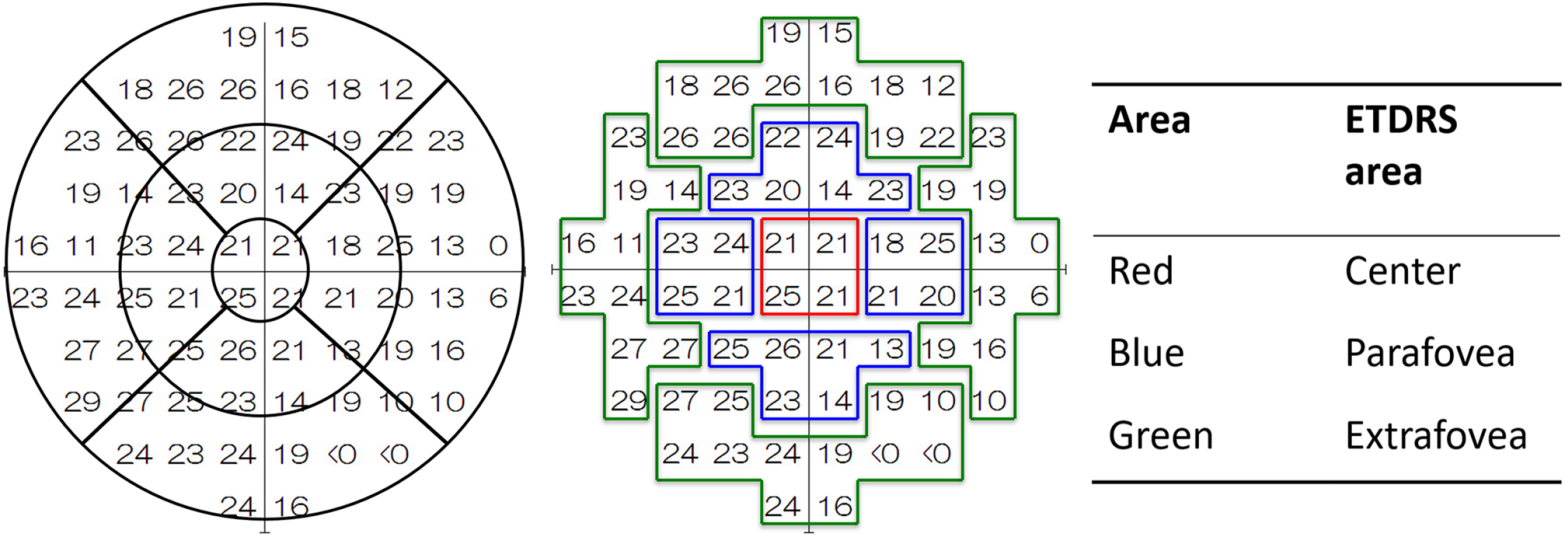
Mapping of VF. All 68 points were assigned to each of the nine regions after ETDRS^40^. The mean retinal sensitivity was calculated in each sector. *OCT* optical coherence tomography.

To analyze each section of the ETDRS grid, we assigned the measurement thresholds for all 68 points to the nine regions of the ETDRS grid and calculated the mean values of points that were assigned to each of the nine regions as a guide (Fig. 3). The OCT analysis region (6 mm) and the 10-2 measurement region were considered to be equivalent; analysis was performed in the range corresponding to each of the nine regions of the ETDRS grid.

### Comparison of RS between BRVO-affected and unaffected areas

**Figure 4:**
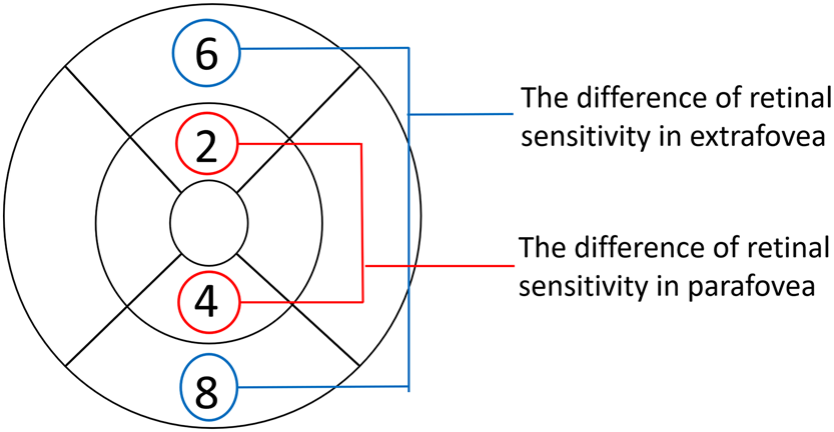
Comparison of the RS between BRVO-affected and unaffected areas. In the parafovea, the difference between areas 2 and 4 was calculated using both B/Y and W/W perimetries. In the extrafovea, the difference between areas 6 and 8 was calculated. *RS* retinal sensitivity, *B/Y* blue on yellow, *W/W* white on white, *BRVO* branch retinal vein occlusion.

In this study, all the patients had been carefully examined by ophthalmologists and confirmed to have BRVO in only one eye. Difference in RS between the defective and nondefective parts was compared using both perimetries. In the parafovea, the difference in RS between the inner–superior and inner–inferior areas was calculated using both W/W and B/Y perimetries. In the extrafovea, the difference between the outer–superior and outer–inferior areas was calculated (Fig. 4). Other areas were excluded in this analysis because BRVO-affected and unaffected areas were mixed.

### Statistical analysis

BCVA was measured in decimal units and converted to logMAR units for statistical analyses. Univariate and multivariate analyses were performed to investigate the correlation between OCT parameters and BCVA, RS_W/W, and RS_B/Y. This analysis was followed by model selection using the AICc index. In a multivariate regression model, degrees of freedom decrease with an increase in the number of variables; hence, the use of model selection methods is recommended to improve the model fit by removing redundant variables, particularly when the number of explanatory variables is large, rather than performing simple multivariate regression analysis^41,42^. Using model selection with AICc, the model with the smallest AICc value was selected as the optimal model. AIC is an established statistical measure used to evaluate the relationship between variables, and AICc is a corrected form of AIC, providing accurate estimation even when the sample size is small^43^. The difference in RS between the BRVO-affected and unaffected areas was compared between W/W and B/Y perimetries using paired Student’s *t* test.

R statistical software version 3.5.2 (The R Foundation for Statistical Computing, Vienna, Austria) was used for all analyses.

## Supplementary information

Supplementary Information.
