# Supplementary material for: Comparison between blue-on-yellow and white-on-white perimetry in patients with branch retinal vein occlusion
Source: Sci Rep. 2020 Nov 17;10:20009. doi: 10.1038/s41598-020-77025-x (PMC7672051; doi:10.1038/s41598-020-77025-x)
Supplement: Supplementary file 1 — Supplementary Information. [file 41598_2020_77025_MOESM1_ESM.pdf]

Comparison between blue-on-yellow and white-on-white perimetry in patients with branch retinal vein occlusion

Kunihiro Azuma[1], Tatsuya Inoue[1, 2], Ryosuke Fujino[1], Nozomi Igarashi[1], Shotaro Asano[1], Yoko Nomura[1], Yohei Hashimoto[1], Keiko Azuma[1], Ryo Asaoka[1], Kazuaki Kadonosono[2], Ryo Obata[1]

1: Department of Ophthalmology, The University of Tokyo, Tokyo, Japan

2: Department of ophthalmology and micro-technology, Yokohama City University, Kanagawa, Japan

3: Department of Ophthalmology, Seirei Hamamatsu General Hospital, Shizuoka, Hamamatsu, Japan

4: Seirei Christopher University, Shizuoka, Hamamatsu, Japan

Supplementary Figure S1 Superimposing retinal sensitivity on the OCTA image.
The representative image of central macular non-perfuison eye. Binarized OCTA image was obtained using ImageJ software, following the guideline. Then retinal sensitivity was superimposed on the OCTA image, using AP-7000 built-in software.
OCTA, optical coherence tomography angiographyThe optical coherence tomography angiography image was inverted to match retinal sensitivity.

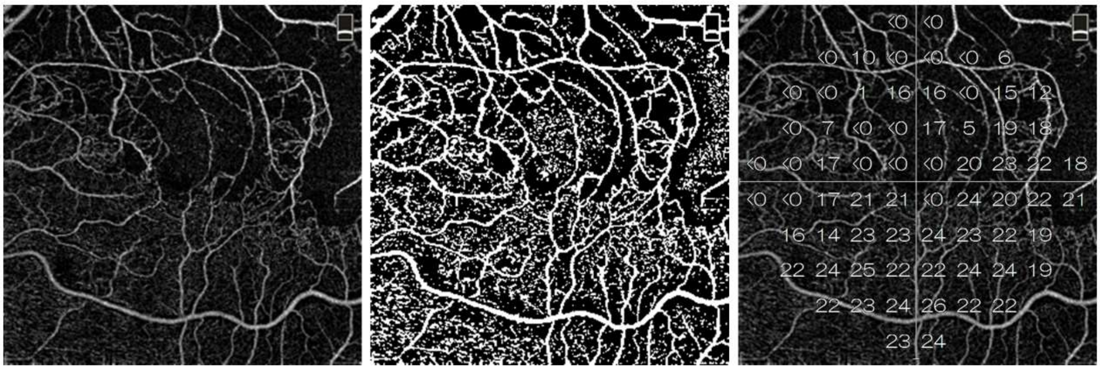

Supplementary Table S1

The correlation between best corrected visual acuity and other parameters

		Univariate			Multivariate		
	Variables	Estimate	SE	P-value	Estimate	SE	P-value
Center	age	0.0029	0.0036	0.44	N.S	N.S	N.S
	VDs	-0.0033	0.0057	0.57	N.S	N.S	N.S
	VDd	-0.0007	0.0049	0.88	N.S	N.S	N.S
	RV	-0.96	1.27	0.46	N.S	N.S	N.S
Parafovea	age	0.0029	0.0036	0.44	N.S	N.S	N.S
	VDs	-0.014	0.0064	0.037*	N.S	N.S	N.S
	VDd	-0.011	0.0057	0.059	N.S	N.S	N.S
	RV	-2.07	0.76	0.011	-2.07	0.76	0.011

SE; Standard error, VDs; Vesssel density in superficial capillary plexus, VDd; Vessel density in deep capillary plexus, RV; Retinal volume, N.S.; not selected (linear model, model selection using the second-order bias corrected Akaike Information Criterion index)
